# A New Approach for Reconstruction of Severe Horizontal Atrophy of the Posterior Mandible Using “The Honeycomb Technique”: A 10–14 Year Follow-Up Retrospective Study

**DOI:** 10.3390/jcm14072246

**Published:** 2025-03-25

**Authors:** Fares Kablan

**Affiliations:** 1 Department of Oral and Maxillofacial Surgery, Galilee College of Dental Sciences, Galilee Medical Center, Nahariya 2210001, Israel; kablanp1@gmail.com; 2 The Azrieli Faculty of Medicine, Bar-Ilan University, Safed 1311502, Israel

**Keywords:** bone augmentation, autogenic bone grafts, allogenic bone substitute, space maintenance, mandibular atrophy

## Abstract

**Background**: Autogenous bone grafting has long been the standard for augmenting bone prior to implant placement in atrophic ridges. However, innovative techniques are continually sought to enhance outcomes. This study introduces the honeycomb technique for horizontal bone augmentation in edentulous posterior mandibular ridges, presenting the methodology and long-term follow-up results of this novel approach. **Methods**: This study includes healthy patients with moderate to severe horizontal atrophy in posterior mandibular regions who underwent bone augmentation using the honeycomb technique and were followed up for a period of 10 to 14 years. The patients had orthoradiographs immediately post-surgery and underwent regular clinical and radiographic evaluations. Computed tomography at four months assessed the bone gain, followed by reentry for implant insertion and evaluation of the bone volume and quality. Fixed prosthesis-supported dental implants were placed four months post-insertion. The survival and success of the dental implants were evaluated based on the acceptable clinical and radiographic criteria. **Results**: A cohort of 23 patients (17 women, 6 men, mean age 47 years) underwent bone augmentation at 39 sites, with follow-up ranging from 10 to 14 years. The procedure demonstrated a 95–100% success rate with minimal morbidity and horizontal bone gain averaging 3–8 mm. Partial graft exposure occurred in two cases but was successfully managed without compromising augmentation. A total of 103 implants were placed in the augmented sites in 37 sites. The long-term survival of the dental implants was confirmed based on clinical and radiographic evaluation, with minimal marginal bone loss observed during the extended follow-up period. **Conclusions**: The honeycomb technique proves effective in horizontal bone augmentation of atrophic ridges in posterior mandibular defects. The satisfactory long-term outcomes validate its potential as a valuable addition to bone augmentation strategies preceding implant placement.

## 1. Introduction

Partial or complete edentulism is most effectively addressed through dental implant therapy, which has demonstrated significant success in modern dentistry [1,2]. However, the successful insertion of dental implants necessitates adequate volume and quality of alveolar bone. Following tooth extraction, horizontal bone loss ranges from 29% to 63%, while vertical bone loss ranges from 11% to 22% within the first six months. This initial phase of resorption is often followed by a gradual yet continuous reduction in bone volume over time [3]. To achieve optimal outcomes, dental implant therapy can be performed at various stages: immediately at the time of extraction, 6 weeks post-extraction (immediate-delayed), or after 4 months of healing (delayed) [4]. For delayed implant placement, socket preservation may be considered to maintain the hard and soft tissue dimensions at the extraction site [5,6]. Conversely, prolonged edentulism can result in significant bone atrophy and alveolar ridge deficiencies. In such cases, ridge augmentation is often necessary to enable the placement of dental implants with optimal length, diameter, and prosthetic positioning. For long-term success, dental implants should be surrounded by 1.5 to 2 mm of bone on all sides [7]. Notably, horizontal bone augmentation in the posterior mandible presents unique challenges due to the limited bone volume and the proximity to vital structures [8].

Various surgical techniques have been proposed to address this issue. Less invasive approaches, such as narrow implants, can often circumvent the need for extensive bone augmentation procedures. According to Grandi et al., narrow implants with diameters ranging from 2.75 to 3.25 mm are suitable for placement in regions where the residual bone width is between 3 mm and 6 mm in the posterior mandible [9]. Esposito reported the use of 3 mm diameter implants as an alternative to horizontal bone augmentation for placing 4 mm diameter implants in partially edentulous patients with a buccolingual crestal bone width of 4 to 5 mm, as measured on CBCT scans. After one year of follow-up following implant loading, the narrow implant approach demonstrated superior outcomes [10]. When narrow implants are not feasible, ridge split procedures may be considered. These techniques are commonly used to augment maxillary alveolar crests but are less frequently applied in the posterior mandible. To perform a ridge split effectively, a minimum ridge width of 3 mm is generally required [11,12]. If the bone width is less than 3 mm, and the alveolar process height is sufficient but the crest is too narrow to support an implant, lateral augmentation is necessary. Various surgical techniques and bone grafting procedures are available to regenerate horizontal alveolar crest dimensions in the edentulous posterior mandible [13,14].

Autogenous bone grafts are considered the gold standard due to their superior biological properties, safety profile, and excellent integration with the recipient site. Common intraoral donor sites for autogenous bone include the chin and retromolar area, each with varying degrees of morbidity and potential complications [15,16]. The ramus has been favored as a donor site due to its lower complication rates, proximity to the recipient site [17], and excellent spontaneous healing capacity [18]. Non-autogenous bone grafts, including allografts, xenografts, and synthetic substitutes, offer several advantages, such as eliminating complications related to autogenous donor sites and providing greater availability. These materials have also been successfully combined with autogenous bone to improve grafting outcomes [19,20,21,22,23].

This article introduces the honeycomb technique (HCT), a novel method for bone augmentation in the posterior mandible with severe horizontal atrophy, characterized by a width of less than 3 mm. The technique combines autogenous bone, rich in living cells, with allogenic particulate bone, which offers virtually unlimited availability. A key innovation of this method is the strategic use of autogenous bone blocks harvested from the mandibular ramus, as detailed in this study.

## 2. Materials and Methods

### 2.1. Patient Selection

This study involved 23 patients (17 women, 6 men) with a mean age of 47 years. All participants were non-smokers in good general health, without chronic diseases or consistent medication use. The patients presented with severe horizontal ridge atrophy, with bone widths ranging from 1.0 to 4.0 mm (mean ± SD: 2.3 ± 0.7 mm). Only patients with a minimum follow-up of 10 years after dental implant loading (range: 10–14 years) were included (Table 1).

All the patients received a detailed explanation of the procedure and signed written informed consent. The study was conducted in accordance with the Declaration of Helsinki and approved by the Ethics Committee of the GMC; NHR-0149-21 (8 February 2022–2 February 2026).

### 2.2. Surgical Protocol

Horizontal bone augmentation using the honeycomb technique was performed at 39 posterior mandibular sites in most areas. Augmentation was performed at two posterior mandibular sites in most cases. The retromolar/ramus area served as the donor site for the bone block used in the honeycomb technique.

The postoperative care instructions included a soft diet for six weeks, antibiotic regimen (Amoxicillin 500 mg/Clindamycin 300 mg, three times daily for 10 days), strict oral hygiene instructions, and a prohibition on the use of removable appliances.

Follow-up examinations were conducted every two weeks. Four months after the procedure, computed tomography scans were performed to assess the bone gain and evaluate the suitability of the newly formed bone for dental implant placement. Reentry procedures were carried out 4 to 5 months post-operation to assess the bone volume and facilitate implant placement. Prosthetic rehabilitation was initiated three months after implant placement and was performed by the patients’ dentists. The long-term follow-up encompassed periodic radiographs to monitor marginal bone loss around the implants.

The patients were instructed on strict oral hygiene protocols and scheduled for periodic follow-up visits with their dentists and dental hygienists, as recommended by the clinical team. Long-term success was closely linked to adherence to these follow-up measures. Clinical and radiographic evaluations were conducted by the patients’ dentists and reported to the author. Marginal bone loss was measured manually on periapical x-rays in both millimeters and percentages. The patients included in this study were monitored over a follow-up period of 10 to 14 years after loading.

The study was conducted in accordance with the Declaration of Helsinki, and approved by the Ethics Committee of the GMC; NHR-0149-21 (8 February 2022–2 February 2026). Written informed consent for publication was obtained from the patients.

### 2.3. Illustration Case (Patient No. 21)

**Figure 1:**
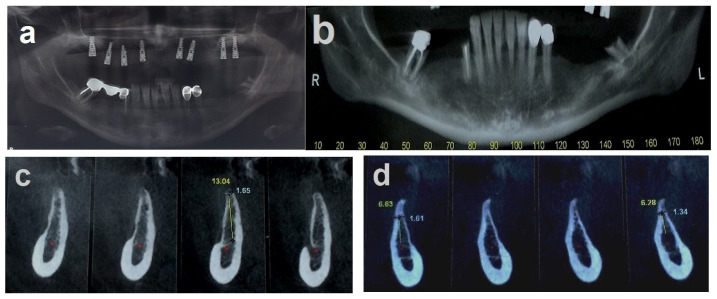
(**a**) Bilateral posterior mandibular edentulism. (**b**–**d**) Computed tomography showing severe horizontal atrophy with knife-edge residual ridges.

A 42-year-old female patient was referred to our clinic for bilateral augmentation of the posterior mandible and dental implant placement. The patient was in good health, presenting with bilateral posterior mandibular edentulism (Kennedy Class I) (Figure 1a,b). The CBCT scan revealed severe horizontal atrophy of both posterior mandibular ridges, with knife-edge residual ridges (Figure 1c,d). Narrow implants or ridge splitting were not viable options. Therefore, bone augmentation was required to achieve the treatment goal. The patient was treated using the honeycomb technique.

### 2.4. Honeycomb Technique Procedure

#### 2.4.1. Donor Site Preparation

Under IV sedation and local anesthesia with a vasoconstrictor, a full-thickness mucoperiosteal flap was elevated, with a mesial releasing incision in the premolar region to expose the recipient site. The flap was extended over the oblique ridge toward the ramus to provide access to the donor site.

#### 2.4.2. Bone Block Harvest

**Figure 2:**
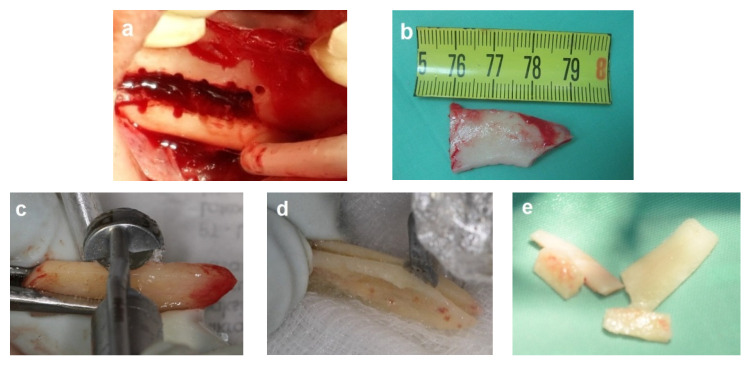
(**a**) The donor site. (**b**) The bone block harvested from the retromolar donor site. (**c**–**e**) The harvested bone block and its subsequent splitting, resulting in thin bone blocks and cortical wedges.

A cortical bone block was harvested from the retromolar area. The dimensions of the bone block—posterior–anterior length and superior–inferior width—were carefully determined and executed through three complete osteotomies: posterior, anterior, and inferior (Figure 2a). The bone block typically exhibits D1 in density, with a thickness of 3–4 mm, a length of 2–3 cm, and a width of 8–12 mm (Figure 2b). Using a MicroSaw (MicroSaw, Dentsply Sirona’s Frios, Dentsply Implants, Mannheim, Germany), the harvested bone block was longitudinally split into two thinner bone plates, then further divided along the same axis to create three to four thin plates (Figure 2c,d). Two plates were set aside, while the remaining ones were transversely split to create multiple thin bone wedges (Figure 2e). This process resulted in two thin cortical bone plates and multiple cortical bone wedges.

#### 2.4.3. Recipient Site Preparation

**Figure 3:**
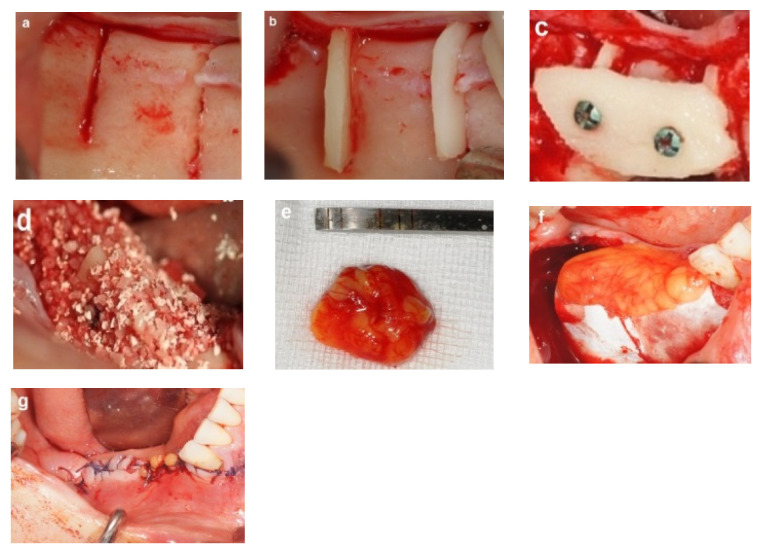
The bone augmentation procedure. (**a**) Grooves created using high-speed bur at the recipient site. (**b**) Insertion and tapping of the cortical bone wedges into the grooves. (**c**) Positioning a thin bone plate over the bone wedges and securing it with screws, forming bone compartments resembling a honeycomb. (**d**) Filling the bone compartments with allogenic particulate bone graft to achieve the desired bone graft volume. (**e**) Free buccal fat tissue graft (BFFG). (**f**) Resorbable membrane and BFFG covering the augmented bone. (**g**) Double-layer tension-free closure of the recipient site.

The augmented bed was carefully prepared by creating grooves or slots using high-speed thin straight burs, low-speed small straight burs, or a piezoelectric device. The number of grooves was determined by the length of the augmented ridge. Ideally, these grooves should traverse the bone completely from buccal to lingual and extend as deeply as possible. However, their depth was limited by proximity to the inferior alveolar nerve, a factor assessed via dental CT scans (Figure 3a). The primary function of these grooves in the honeycomb technique is to provide both biological integration and mechanical stabilization for the bone wedges.

#### 2.4.4. Augmentation Procedure

1. Bone wedges were positioned into the prepared grooves at the recipient site, ensuring that each wedge fitted securely within its respective groove. Once properly adapted, each wedge was gently tapped into place using a flat-edged cylindrical instrument and a hammer. Stability was verified by attempting to dislodge each wedge, and any unstable wedge should be replaced (Figure 3b).
2. A thin cortical bone plate was secured laterally over the bone wedges using screws, creating multiple bone compartments resembling a honeycomb structure (Figure 3c). Sharp edges were trimmed to avoid soft tissue trauma. The horizontal dimensions of the augmented site are determined by the combined dimensions of the bone wedges and the laterally fixed cortical bone block.
3. The compartments were filled with allograft particulate bone (Figure 3d), covered with a collagen membrane (Geistlich Bio-Gide, Geistlich Pharma, Switzerland), and covered with a free buccal fat pad graft that was secured with sutures (Figure 3e,f). The free buccal fat pad graft enhances the flap closure and provides soft tissue augmentation.
4. The flap was closed using tension-free sutures (Figure 3g).

#### 2.4.5. Postoperative Management

**Figure 4:**
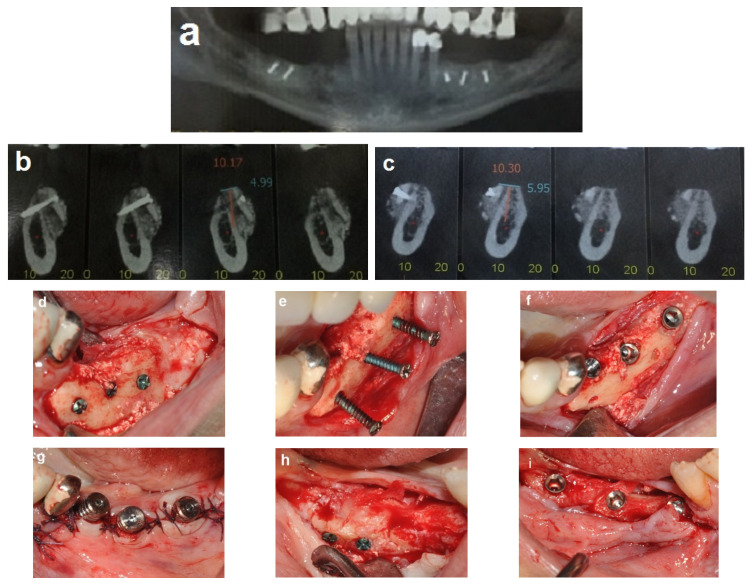
Reentry at 4 months. (**a**–**c**) CBCT post-op demonstrating significant horizontal bone gain. (**d**) Left-side view, showing excellent integration of the bone blocks and the width of the new bone demonstrated by the length of the removed fixation screws (**e**). (**f**,**g**) Placement of three implants on the left side. (**h**) New bone volume on the right side. (**i**) Placement of three implants on the right side.

**Figure 5:**
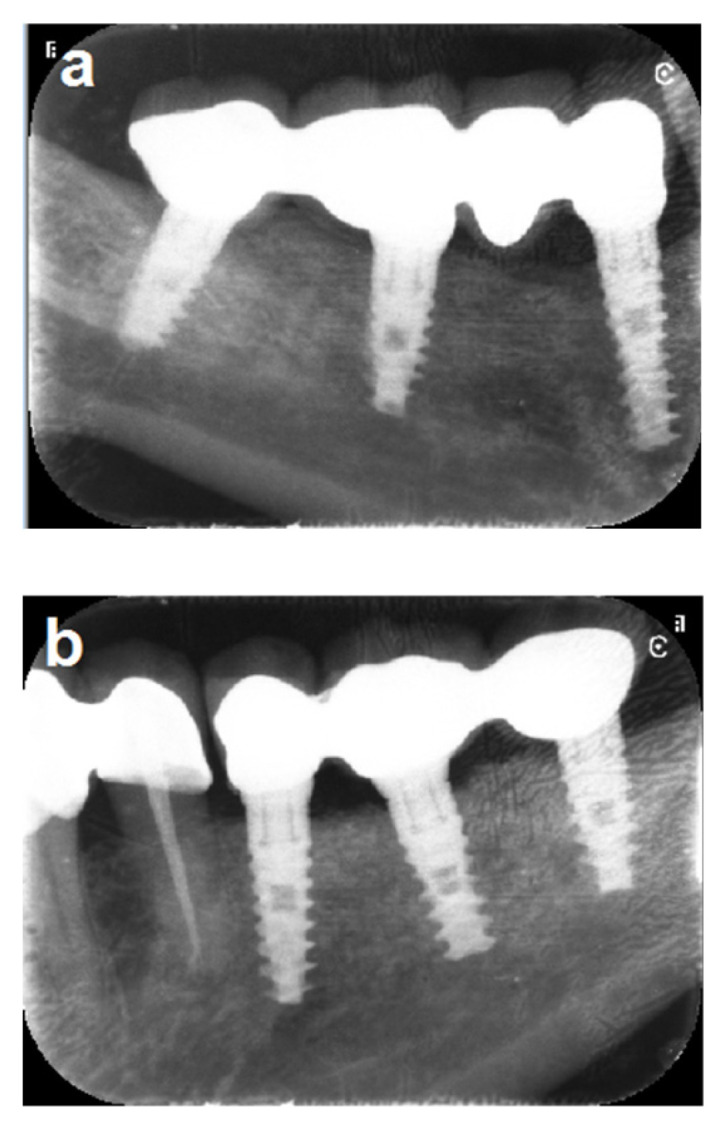
Follow-up. (**a**,**b**) Ten-year follow-up post-loading, showing the implant at the recipient site with no marginal bone loss.

Follow-up was scheduled three times every two weeks, then monthly. Partial exposure of the graft occurred at four weeks on the right side but was successfully managed without compromising the augmentation outcome. A CBCT scan was obtained after four months to assess the bone gain (Figure 4a–c). Dental implants were placed 4 months after the procedure (Figure 4d–i). A fixed prosthesis was delivered four months after the implant placement. This case was followed for 10 years (Figure 5).

### 2.5. Case Presentation

#### 2.5.1. Case 1 (Patient No. 20)

**Figure 6:**
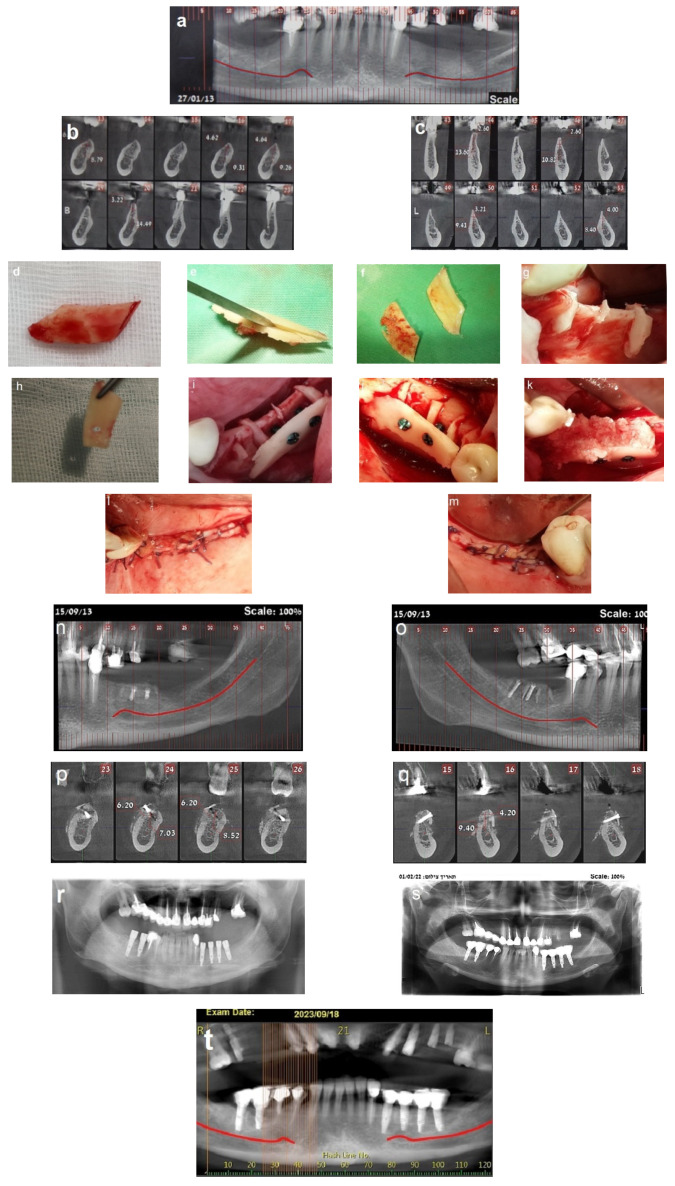

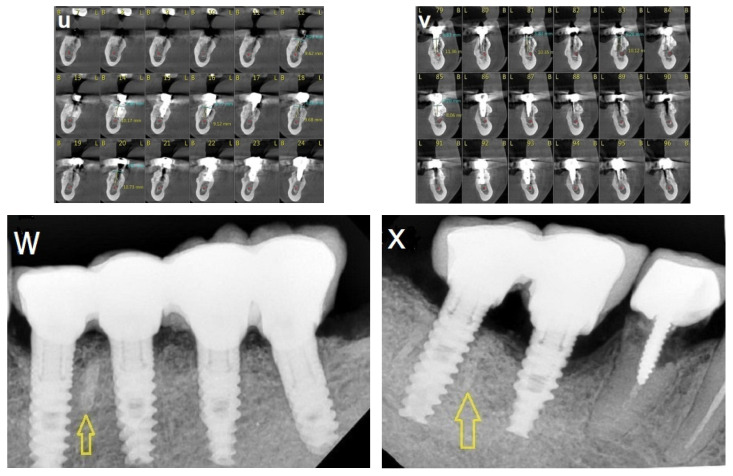
(**a**) Bilateral posterior mandibular edentulism. (**b**,**c**) Computed tomography showing severe horizontal atrophy with knife-edge residual ridges. (**d**) The bone block harvested from the retromolar donor site. (**e**,**f**) The harvested bone block and its subsequent splitting, resulting in thin bone blocks and cortical wedges. (**g**) The bone augmentation procedure: grooves were created using high-speed at the recipient site, followed by the insertion and tapping of the cortical bone wedges into the grooves. (**h**,**i**) A thin bone plate is positioned over the bone wedges and secured with screws, creating bone compartments resembling a honeycomb. (**j**) Right side. (**k**) The bone compartments are filled with allogenic particulate bone graft to achieve the desired graft volume on the right side. (**l**,**m**) Double-layer, tension-free closure of the recipient sites performed using BFFGs. (**n**–**q**) CBCT scan, four months postoperatively show significant bilateral horizontal bone gain. (**r**) Two implants were placed on the right side and four implants on the left side. (**s**) Panoramic view at 8-year follow-up. (**t**–**v**) CBCT scans taken 10 years after implant placement reveal stable augmented bone levels. (**w**) Eleven-year follow-up periapical x-ray of the left side, demonstrating no significant marginal bone loss. (**x**) 3 mm of marginal bone loss observed at the mesial implant. The yellow arrows show the cortical wedges that remain visible at the recipient sites.

A 36-year-old woman was referred for bone augmentation and dental implant placement in the atrophic posterior mandibular ridges. The examination revealed partial edentulism of the mandible, with missing posterior teeth on both the right and left sides (Figure 6a). Computed tomography confirmed a significant horizontal bone deficit in the posterior mandibular regions bilaterally (Figure 6b,c). A two-stage treatment approach was planned. In the first stage, the honeycomb bone (HCT) technique was employed to augment the right and left posterior mandibular regions.

In May 2013, a cortical bone block was harvested from the right retromolar area (Figure 6d). The harvested block was then split, yielding two thin bone blocks and multiple bone wedges (Figure 6e,f). The recipient sites were prepared by creating grooves, into which the bone wedges were inserted in a stable position bilaterally (Figure 6g). Two thin bone blocks were then secured over the bone wedges using screws. This configuration resulted in multiple bone compartments resembling a honeycomb structure (Figure 6h–j). After trimming any sharp edges, the compartments were filled with particulate allograft bone substitute, achieving the desired final bone volume (Figure 6k). The augmented sites were then covered with a resorbable membrane and a free buccal fat pad graft (BFFG) before being closed in a tension-free manner (Figure 6l,m). The healing process was uneventful throughout the follow-up period. A CBCT scan performed four months postoperatively, in September 2013, confirmed significant horizontal bone gain of 4–6 mm (Figure 6n–q). Upon reentry under local anesthesia, the newly formed bone volume was evident, with the bone wedges fully integrated into the regenerated bone mass. Six dental implants were successfully placed (Figure 6r). After three months, all the implants had achieved full osseointegration, allowing for final prosthetic rehabilitation. This case has been followed for 11 years. A panorex obtained at 8 years showed implants without marginal bone loss (Figure 6s), and a CBCT scan obtained in September 2023 demonstrated stable bone volume 10 years post-surgery, with no resorption of the augmented bone blocks (Figure 6t–v). Periapical radiographs taken in July 2024 further confirmed the long-term stability of the outcomes and implant survival. At this time, the first implant on the right side showed a 3mm marginal bone loss, possibly due to food impaction from a decayed adjacent premolar. The remaining five implants exhibited no signs of marginal bone loss. The cortical wedges remained visible at the recipient sites in both figures (Figure 6w,x).

#### 2.5.2. Case 2 (Patient No. 12)

**Figure 7:**
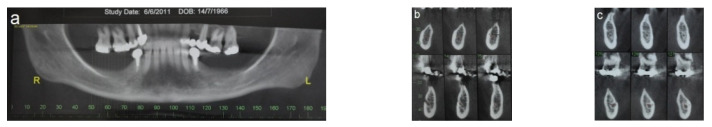

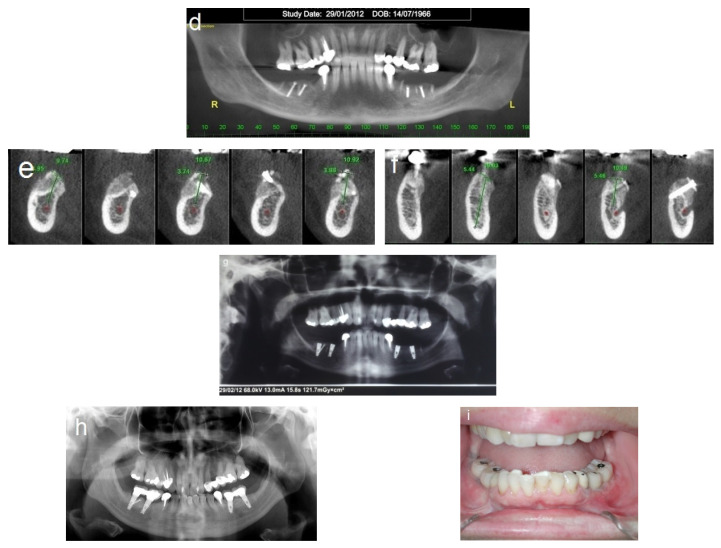
(**a**–**c**) CBCT scan images reveal severe horizontal atrophy in the molar regions bilaterally. (**d**–**f**) CBCT scan images taken after honeycomb bone grafting demonstrate significant bone gain. (**g**); Implants, (**h**,**i**) images taken 13-year follow-up after implant loading.

A 47-year-old healthy female patient was referred to our clinic for bilateral augmentation of the posterior mandible and dental implant placement. She was treated using the honeycomb technique and underwent bilateral augmentation of the posterior mandibular ridges. Figure 7 presents the CBCT scans before and after the augmentation procedure (Figure 7a–f), as well as the stable outcomes observed after 13 years of follow-up (Figure 7g,h).

## 3. Results

### 3.1. The Healing Process

Healing of the surgical sites was uneventful in 21 patients across 36 treated sites. However, in two patients, partial graft exposure due to minor wound dehiscence occurred 3–4 weeks postoperatively at the right-side augmentation site. This was managed conservatively by trimming the exposed bone, which was followed by spontaneous soft tissue healing without significant bone loss. After a healing period of 4–5 months, CBCT scans were performed for all the patients to assess the bone gain and new bone dimensions.

### 3.2. Bone Gain and Implant Placement

The CBCT analysis revealed a horizontal bone gain ranging from 3 to 8 mm, with an average of 5.1 mm. No additional bone augmentation was required. One patient (No. 14), who had undergone augmentation at two sites, was lost to follow-up due to relocation and did not receive dental implants at our clinic.

The mean pre-treatment bone width was 2.3 ± 0.7 mm. Following the honeycomb technique, the mean post-treatment bone width increased to 7.4 ± 1.2 mm, with a mean bone gain of 5.1 ± 1.3 mm (range: 3.0–8.0 mm).

A paired *t*-test revealed a statistically significant increase in bone width post-treatment compared to baseline (t = 12.45, *p* < 0.001).

The Wilcoxon signed-rank test showed a significant increase in bone width post-treatment (Z = −5.24, *p* < 0.001).

### 3.3. Implant Placement and Follow-Up

A total of 103 dental implants, with diameters ranging from 3.3 to 4.2 mm (average: 3.8 mm), were placed at 37 augmented sites in 22 patients (Table 2). One implant failed during the uncovering stage and was successfully replaced after three months (Patient No. 13). Patients were subsequently referred to their general dentists for fixed prosthetic rehabilitation three months after implant placement.

**Figure 8:**
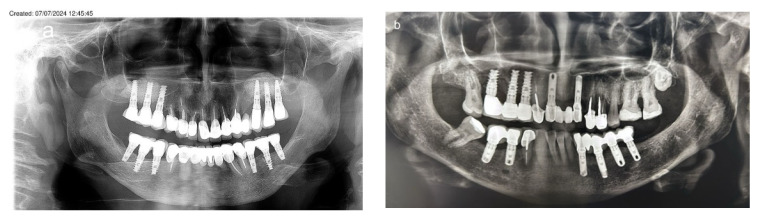
(**a**) Patient No. 1; follow-up 14 years. (**b**) Patient No. 5; follow-up 14 years.

Patients were instructed on strict oral hygiene protocols and scheduled for periodic follow-up visits with their dentists and dental hygienists, as recommended by the clinical team. Long-term success was closely linked to adherence to these follow-up measures. Clinical and radiographic evaluations were conducted by the patients’ dentists and reported to the author. The patients included in this study were monitored for a follow-up period of 10–14 years. Examples include Patient No. 1 (14-year follow-up; Figure 8a) and Patient No. 5 (14-year follow-up; Figure 8b).

### 3.4. Implant Success Rate

**Chart 1:**
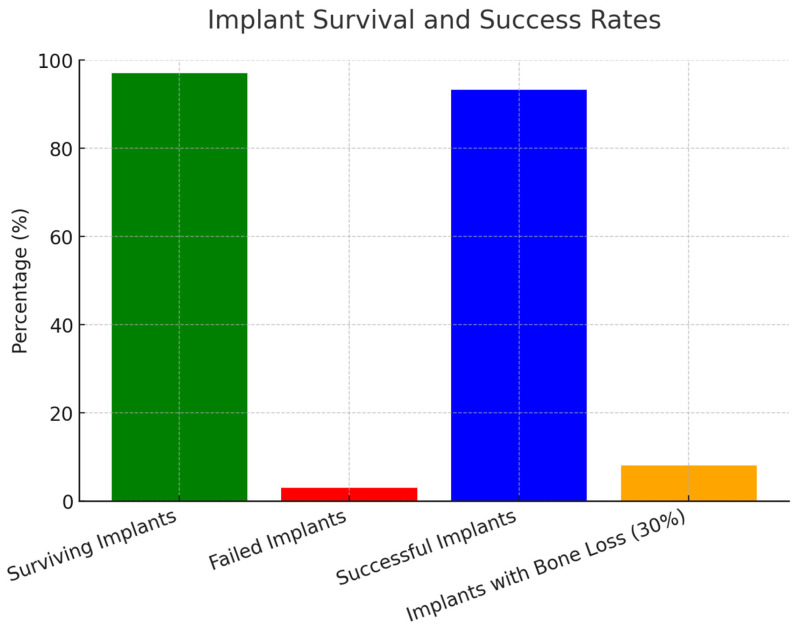
The bar chart illustrates the percentage of surviving implants (97%), failed implants, successful implants (93.2%), and implants with marginal bone loss of 30%.

Dental implants placed in sites augmented with the honeycomb technique (HCT) exhibited a 93.2% success rate over a follow-up period of 10–14 years (Table 3 and Chart 1).

**Figure 9:**
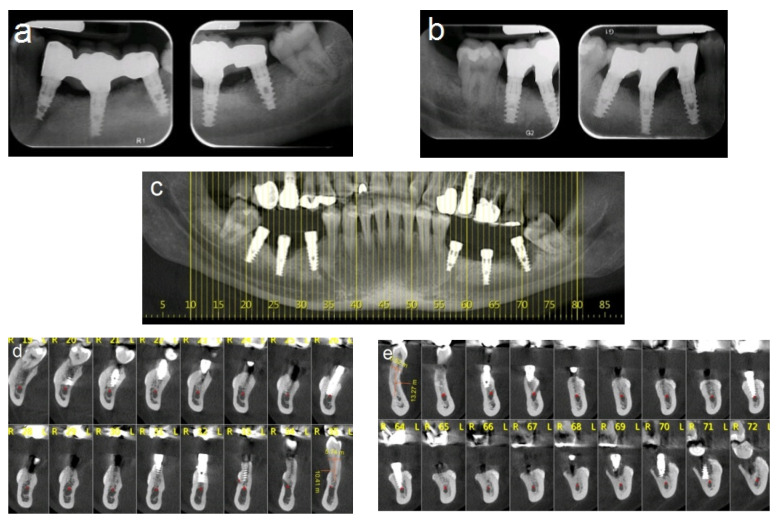
(**a**,**b**) Ten-year follow-up periapical x-rays reveal severe bone loss involving the distal implant on the left side, along with 3 mm of marginal bone loss at the middle implant on the right side. (**c**–**e**) The images demonstrate stable bone grafts despite the severe marginal bone loss at the distal implant on the left side.

**Figure 10:**
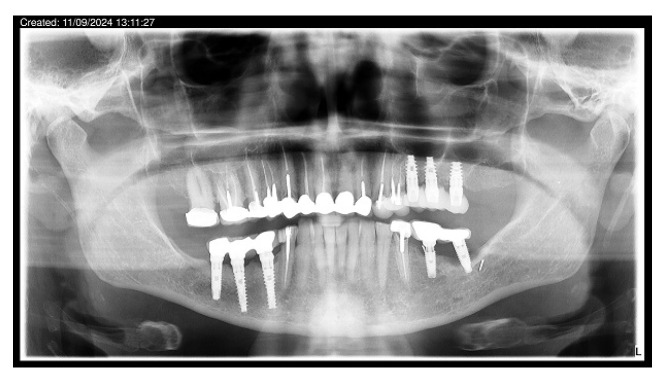
Panoramic x-ray taken 13 years after implant loading reveals severe marginal bone loss involving the distal implant, along with a fracture of the mesial implant hex.

In one patient (Patient No. 19), one of three implants at the left mandibular site failed after 10 years. The failed implant was removed and replaced four months after particulate bone grafting, leading to successful fixed prosthetic rehabilitation with the remaining two implants. The same patient exhibited a 3 mm marginal bone loss on the right side (Figure 9). Another patient (Patient No. 6) experienced severe bone loss affecting the distal implant, along with a fracture of the mesial implant, after 13 years (Figure 10).

**Figure 11:**
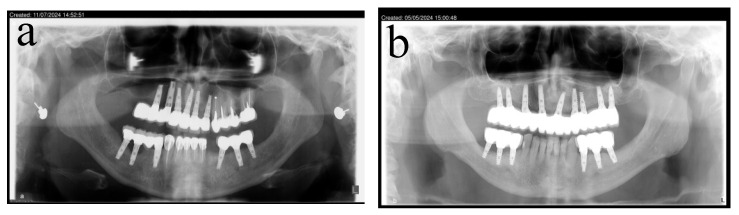
(**a**) A panoramic x-ray taken 13 years after implant loading demonstrates stable outcomes across five implants, though the middle implant on the right side exhibits 3 mm marginal bone loss. (**b**) A panoramic x-ray taken 11.5 years after implant loading shows stable outcomes for five implants, with 3 mm marginal bone loss affecting the distal implant on the right side.

Five implants in five patients exhibited a 3 mm bone loss; however, they remained asymptomatic and were successfully maintained with regular professional cleaning (e.g., Patient No. 7, Figure 11a, and patient No. 18, Figure 11b).

Overall, seven implants did not meet the full success criteria, resulting in a 93.2% implant success rate over the 10–14 year follow-up period.

### 3.5. Implant Survival Rate

**Chart 2:**
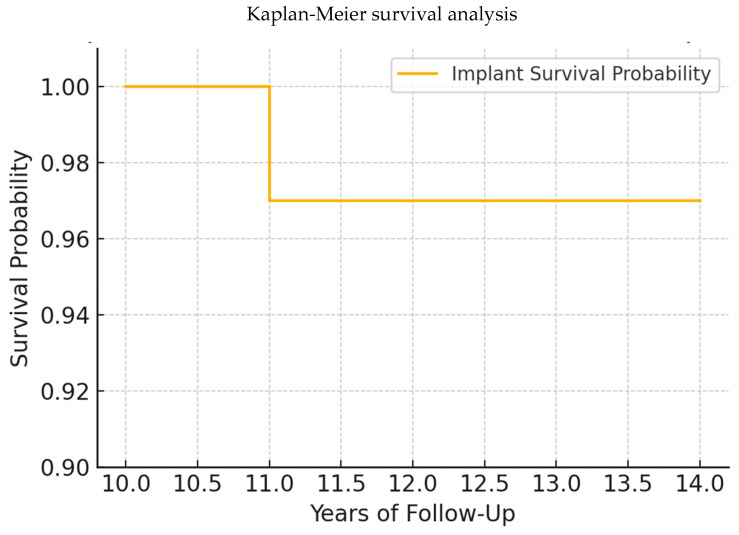
Kaplan–Meier survival analysis for dental implants over a follow-up period of 10 to 14 years.

Out of 103 implants, three implants failed over the course of 10–14 years, yielding a 97% implant survival rate during this period (Table 3, Chart 1 and Chart 2).

Further follow-up of the patients included in this study, adhering to the same prophylactic regimen is considered to evaluate long-term outcomes over an extended period.

## 4. Discussion

Long-term edentulism in the posterior mandibular region is a common clinical scenario. Both patients and treatment teams generally prefer less invasive surgical procedures for implant placement. Narrow implants have gained popularity in recent years and are considered a predictable option when the residual bone width is greater than 4–5 mm [10]. Ridge splitting is another effective technique that eliminates the need for a donor site. This method is suitable when the residual bone width exceeds 3 mm and ideally ranges between 4–5 mm [24]), depending on the specific approach. In cases of severe ridge atrophy, where the bone width is reduced to less than 3 mm and the deficiency significantly affects ridge height, lateral bone grafting is typically required to achieve successful implant placement.

Autogenous bone grafts have long been considered the gold standard for reconstructing alveolar defects due to their unique combination of osteoinductive, osteogenic, and osteoconductive properties. Harvesting bone blocks from the mandibular ramus is a widely accepted technique, offering several advantages, including low morbidity and the close proximity between the donor and recipient sites, particularly in posterior mandibular reconstructions [13,16,17]. Additionally, intraoral block grafts demonstrate reduced long-term surface resorption, contributing to the enhanced stability and longevity of osseointegrated implants [25].

Pikos in 2005 reported that a single mandibular ramus donor site could provide sufficient bone volume to restore a three-tooth segment and achieve 3–4 mm of horizontal or vertical bone regeneration [26]. Since ramus bone is primarily composed of cortical bone, it may be more resistant to revascularization. To overcome this limitation and promote better vascular integration, Khoury recommends splitting the bone block—a technique that enhances revascularization and improves healing outcomes [13,27]. Recent data suggest that using mandibular bone blocks following the split bone block technique for 3D reconstruction in the posterior mandible demonstrates long-term predictability [28]. The author described the wedge technique for three-dimensional bone augmentation, which is based on the same principle as the split bone block technique. This method involves dividing a harvested bone block from the retromolar area into multiple thin cortical bone segments, known as bone wedges [29].

This approach significantly increases the available surface area for augmentation within the oral cavity, allowing a single bone block from the retromolar area to be used at multiple augmentation sites. The thin structure of the bone wedges further enhances revascularization, supporting improved graft integration and healing. In this report, histological analysis demonstrated the presence of vital osteocytes throughout the entire bone wedge, with osteoblasts and osteoclasts observed along its periphery. Notably, no necrotic areas were identified within the bone wedge, indicating its vitality and active remodeling process [30]. In contrast, clinical studies utilizing a full bone block graft have reported a relatively low presence of vital bone cells, even in cases where graft healing proceeds without complications [31]. Based on the same biological principles, the honeycomb technique is a modification of the wedge technique. It is specifically designed for horizontal augmentation in severely atrophic posterior mandibular sites with a bone width of less than 3 mm, providing a promising and effective solution for such reconstructions. This technique involves splitting a single harvested block into three to four thin cortical bone plates, each measuring 0.5 to 1 mm in thickness, following Khoury’s split bone block (SBB) technique [13]. One or two of these thin plates are preserved, while the remaining are transversely split to create bone wedges, with their width determined by the required amount of lateral bone grafting. The grooves prepared at the recipient site serve two essential functions: they mechanically secure the cortical bone wedges in place and enhance the bone-to-bone contact area, promoting better integration and stability. The thin structure of the cortical bone wedges facilitates early revascularization compared to a standard full bone block graft. Their placement within the prepared grooves increases the bone-to-bone contact surface area, leading to faster integration and improved graft stability. The lateral placement of an additional thin bone block helps achieve the desired final width for horizontal augmentation, creating a stable bony compartment while preventing deformation. This “honeycomb” configuration, formed through the augmentation method, determines the final graft volume. By converting a one-wall defect into a four-wall structure, the technique enhances graft–host interaction, aligning with findings reported in the literature [32]. Filling the honeycomb compartments with particulate bone allograft substitutes enhances graft incorporation at the recipient site while preserving autologous bone. The combination of autogenous and non-autogenous bone grafting materials is widely used and well-documented in the literature [21,22,33].

Dehiscence at bone-augmented sites is a well-documented complication in these procedures, primarily due to the difficulty of achieving a tension-free flap closure [34,35]. Tension-free closure remains a critical factor in the success of bone grafting. To address this challenge, the author consistently employs a buccal fat pad-derived free fat graft (BFFG) for tension-free closure and simultaneous soft tissue augmentation at bone augmentation sites [36]. This technique effectively minimizes soft tissue dehiscence while offering additional protection to the underlying bone graft. The author was the first to investigate and report the clinical and histologic healing process of a BFFG after maxillofacial surgery. BFFG has been shown to heal by rapid epithelialization of the exposed regions and simultaneous fibrosis of the graft that proceeds from immature fibrosis to full maturation after four months [37]. Since 2011, it has been established that fat tissue from the buccal fat pad can survive and heal well, serving as an excellent tissue graft for various defects in the oral cavity [38,39,40,41,42,43].

A recent systematic review by Smeets et al. evaluated horizontal bone augmentation techniques in the mandible using various grafting materials. The authors reported horizontal bone gains ranging from 3.2 mm to 5.7 mm, with a mean increase of 4.8 mm across seven of the eight included studies [14]. Similarly, Barbu et al. reported an average horizontal bone gain of 5.099 mm with autogenous bone grafts and 5.23 mm when combined with platelet-rich fibrin [44]. The horizontal bone gain achieved using the honeycomb technique (HCT) ranged from 3 mm to 8 mm, with a mean of 5.1 mm. These outcomes align with previously reported findings in the literature and have remained stable over a follow-up period of 10 to 14 years. Notably, among studies on lateral bone augmentation of posterior mandibular ridges, the present study reports the longest follow-up duration to date.

According to Smeets et al., the survival rate of dental implants in the included studies exceeded 92.5% over a follow-up period ranging from 18.9 to 37 months. However, none of these studies reported implant success rates [14]. Several studies assessing dental implants placed in ridges augmented with mandibular ramus blocks have documented survival rates between 93% and 99%, with follow-up periods ranging from 19 months to 6 years [45]. Sethi and Kaus reported a survival rate of 98.3% after 77 months [46]. A systematic review by Clementini et al. (2011) examined the success rates of dental implants placed in areas regenerated with autologous bone grafts. The authors found that the success rates of implants placed in onlay graft-regenerated ridges ranged from 72.8% to 97%, with follow-up periods varying from 6 months to 10 years [47], representing the longest follow-up period reported in the literature. In comparison, implants placed using the HCT technique demonstrated a survival rate of 97% and a success rate of 92.2% over a follow-up period of 10 to 14 years post-loading.

This technique proves to be an effective approach for horizontal bone augmentation in atrophic posterior mandibular ridges, demonstrating long-term stability. To further validate this technique, several measures should be considered. Incorporating a control group with alternative augmentation methods would allow for direct comparison. Conducting a randomized controlled trial (RCT) would help minimize selection and confounding biases, ensuring more robust results. Blinded outcome assessments should be implemented to reduce measurement bias. Additionally, performing a detailed subgroup analysis would help determine whether patient-related factors, such as age, bone quality, and systemic conditions, influence the outcomes.

## 5. Conclusions

The honeycomb technique (HCT) presents a reliable, biologically driven approach for horizontal bone augmentation in the posterior mandible. By enhancing revascularization and maximizing bone-to-bone contact, this technique supports stable graft integration and long-term implant success. Its promising long-term outcomes highlight its potential as a valuable addition to bone augmentation strategies prior to implant placement. However, further studies are recommended to confirm its efficacy and refine its application.

## Figures and Tables

**Table 1:** Patient demographics, augmentation details, and follow-up information.

Patient No	Site No	Age	Gender	Augmentation Site	Available Bone (mm)	Donor Site	Follow-Up in Years Since Implant Loading
1	1 2	53	F	35–37 45–47	1.5.0–2.0 2.5–3.5	RT	14
2	3 4	57	M	34–37 43–47	2.0–3.5 1.5–2.0	RT & LT	14
3	5 6	52	F	36–37 46–47	2.0–3.0 2.0–3.5	RT	14
4	7	46	F	44–47	1.5–3.0	RT	14
5	8 9	38	F	32–37 45–46	2.5–3.0 2.0–4.0	RT & LT	14
6	10	46	F	36–37	1.5–3.0	LT	13.5
7	11 12	44	F	34–37 44–47	1.0–3.0 1.0–3.0	RT & LT *	13
8	13	43	F	45–47	2.0–3.5	RT	13
9	14 15	53	M	35–37 45–47	1.5–3.0 1.5–3.0	RT	13
10	16 17	61	M	34–37 45–47	1.5–3.5 1.5–3.0	RT	13
11	18	28	F	36–37	2.0–3.0	LT	13
12	19 20	47	F	36–37 46–47	2.0–3.0 1.5–3.0	RT	13
13	21	62	F	35–37	1.5–3.5	LT	13
14	22 23	53	F	35–37 46–47	1.5–3.0 1.5–3.0	LT	-
15	24 25	56	F	36–37 46–47	2.0–2.5 1.5–2.5	LT	12.5
16	26 27	62	F	34–37 46–47	2.0–4.0 2.5–4.0	RT	12.4
17	28 29	56	M	34–37 44–47	1.5–2.0 1.5–3.0	RT	12
18	30 31	47	M	35–37 45–47	1.5–3.0 1.5–2.5	RT	11.8
19	32 33	37	F	35–37 45–47	2.0–3.0 2.0–3.0	RT	11
20	34 35	36	F	34–37 46–47	2.0–3.0 2.0–3.0	RT	11
21	36 37	42	F	36–37 44–47	1.5–3.0 1.5–3.0	RT	10
22	38	28	M	44–46	1.5–3.0	RT	10
23	39	42	F	44–47	1.5–3.0	RT	10

* The patient underwent augmentation I the anterior maxilla at the same surgery.

**Table 2:** Horizontal bone gain and number and dimensions of the placed implants.

Patient No.	Site No	Horizontal Bone Gain		Implants		
		Right Side	Left Side	No.	Length	Diameter
1	1, 2	3.0–5.0	4.0–6.0	6	10–11.5	3.3–4.2
2	3, 4	3.0–5.5	4.0–5.0	7	10–13	3.3–3.75
3	5, 6	3.0–4.5	3.0–5.0	2	11.5	3.75
4	7	4.0–6.0	-	3	10–13	4.2–3.74
5	8, 9	5.0–6.0	3.5–5.0	6	11.5–13	4.2–3.75
6	10	-	4.0–6.0	3	10–11.5	3.75
7	11,12	5.0–8.0	5.0–8.0	6	10–13	3.73–4.2
8	13	3.0–4.0	-	3	11.5	3.75
9	14, 15	4.0–6.0	4.0–8.0	6	11.5–13	3.75–4.2
10	16, 17	3.0–5.0	3.0–5.0	6	13	3.75
11	18	-	3.5–4.5	2	11.5	3.75
12	19, 20	4.0–6.0	4.0–6.0	4	11.5	3.75
13	21	-	3.0–6.0	3	11.5	3.75
14 *	22, 23	-	-	-	-	-
15	24, 25	3.0–5.0	4.0–8.0	4	10–11.5	3.75–4.2
16	26, 27	3.0–5.0	4.0–6.0	6	11.5–13	3.75–4.2
17	28, 29	4.0–6.0	3.0–6.0	7	10–13	3.75
18	30, 31	3.0–5.0	3.0–6.0	6	10–13	3.75
19	32, 33	4.0–6.0	3.0–4.0	6	10–11.5	3.75
20	34, 35	5.0–8.0	4.0–7.0	6	11.5–13	3.75–4.2
21	36, 37	3.0–7.0	4.0–8.0	6	11.5–13	3.75–4.2
22	38	4.0–7.0	-	2	11.5	4.2
23	39	-	3.0–6.0	3	11.5	3.75

* This patient was excluded due to relocation.

**Table 3:** Implant survival and success rates with detailed finding during 10–14 years of follow-up.

Total number of placed implants: 103 Total number of failed implants: 3/103: Implant survival rate: 97% Total number of implants with marginal bone loss of 30%: 5/103. Implant success rate: 7/103: 93.2%

## Data Availability

The required data can be obtained from the patients’ files.
